# Anemia and malnutrition in indigenous children and adolescents of the Peruvian Amazon in a context of lead exposure: a cross-sectional study

**DOI:** 10.3402/gha.v7.22888

**Published:** 2014-02-13

**Authors:** Cynthia Anticona, Miguel San Sebastian

**Affiliations:** 1 Division of Epidemiology and Global Health, Department of Public Health and Clinical Medicine, Umeå University, Umeå, Sweden; 2 Fundación Cayetano Heredia, Casa Honorio Delgado, Lima, Perú

**Keywords:** anemia, malnutrition, indigenous, Peruvian Amazon, lead exposure

## Abstract

**Background:**

Indigenous children and adolescents of the Peruvian Amazon live in precarious conditions that could increase the risk of malnutrition. A particular problem in the Corrientes river communities is the high exposure to lead among children and adolescents.

**Objective:**

This study aimed to determine the nutritional status of children and adolescents in indigenous communities in the Corrientes river basin and examine risk factors for anemia, stunting, underweight, and wasting.

**Design:**

This was a cross-sectional assessment in children and adolescents aged 0–17 years from six communities (n=330). Data collection included measurement of hemoglobin levels, anthropometrics, blood lead levels (BLLs); a parental questionnaire including demographic and dwelling information; parents’ occupation; and the child's duration of breastfeeding and food consumption. Analysis included univariate, bivariate, and logistic regression.

**Results:**

Overall, anemia prevalence was 51.0%, stunting (proxy for chronic malnutrition) 50.0%, and underweight 20.0%. Bivariate analysis showed that anemia and underweight prevalence was higher in the 0–4 years group (*p*<0.05). No association was found between anemia, stunting, or underweight with gender, community exposure to oil activity, or consumption of river water. Stunting prevalence was higher in the group whose BLLs were >5 µg/dL (*p*<0.05). In the logistic regression analysis, no variable was associated with anemia or underweight. The group 5–11 years and >12 years had 1.9 and 3.1 times higher risk of stunting than the group under five years, respectively. Children and adolescents with BLLs >5 µg/dL had twice the risk of stunting compared to those with lower BLLs.

**Conclusions:**

Half of the study population was found with anemia and stunting. Anemia was more prevalent in the 0- to 5-year age group and stunting in the 12- to 17-year group. The association between stunting and BLLs might be attributed to a direct effect of lead on human growth. Also, poor nutrition and other socioeconomic-related factors may contribute to the simultaneous existence of stunting and elevated BLLs.

Anemia and chronic childhood malnutrition constitute some of the major public health problems in Latin America and the Caribbean. They affect children's growth and development and increase the risk of morbidity and mortality. Around 22 million preschoolers and 9 million children under five in Latin America and the Caribbean suffer from anemia and chronic malnutrition, respectively (1, 2).

In all Latin American countries, the indigenous population is often one of the most disadvantaged groups. Indigenous children and adolescents generally live in precarious conditions and have higher rates of mortality and morbidity than the non-indigenous (3–6).

Peru has one of the largest indigenous populations in Latin America, accounting for 12.0% of the total population below 18 years (8,410,904) (7). Despite the general advancements in children's health in the past decade (i.e. decreasing infant and child mortality rates from 43 and 59 per thousand live births in 1996 to 21 and 29 per thousand in 2004–2006, respectively), the nutritional profile is still worrisome. By 2012, 32.9% of the Peruvian children under five had anemia and 18.0% had chronic malnutrition (8). These figures vary widely among regions, showing a more dramatic nutritional status for indigenous groups in the Amazon region, likely related to 1) their high poverty rates (86.0% are poor and 46.0% are extremely poor), 2) diverse environmental exposures and social conflicts, and 3) their limited access to health care services and education (7, 9). This appears to be the case of the indigenous communities in the Corrientes river basin, where industrialization related to oil exploitation has developed successfully.

The Corrientes river basin has been an active site for oil extraction for more than 40 years to date, suffering serious environmental and social impacts (10) that may affect nutritional status. A particular problem of these communities is the presence of elevated blood lead levels (BLLs) among children and adolescents. In 2006, the Ministry of Health conducted an evaluation in 74 subjects (0–17 years) from seven communities and reported that 66.0% had BLLs ≥10 µg/dL. Data from a concurrent analysis of water and sediments did not permit making any conclusion about the source of exposure (11, 12).

In the period 2009–2011, a field investigation to examine the sources, risk factors, and pathways for lead exposure in children was conducted in the Corrientes communities. The results indicated that the main pathway of exposure was the contact with pieces of lead in practices related to the artisanal manufacture of fishing sinkers. In addition, the communities near oil installations had better accessibility of lead cables and other industrial wastes from which they collected pieces of lead (13).

The present study used part of the data collected during the field investigation (anthropometric indicators and hemoglobin levels) in order to assess the nutritional status of indigenous children and adolescents from the Corrientes river basin and to examine potential risk factors for anemia, stunting, underweight, and wasting.

This paper draws upon two prior focuses of concern in the current national agenda of Peru, nutritional problems among children under five and optimal health for indigenous people (United Nations Declaration on indigenous peoples rights in 2007) (14, 15). Therefore, information on their nutritional profile at macro and local levels, as well as the associated factors will help to guide policymaking.

## Methods

### Setting and population

**Fig. 1:**
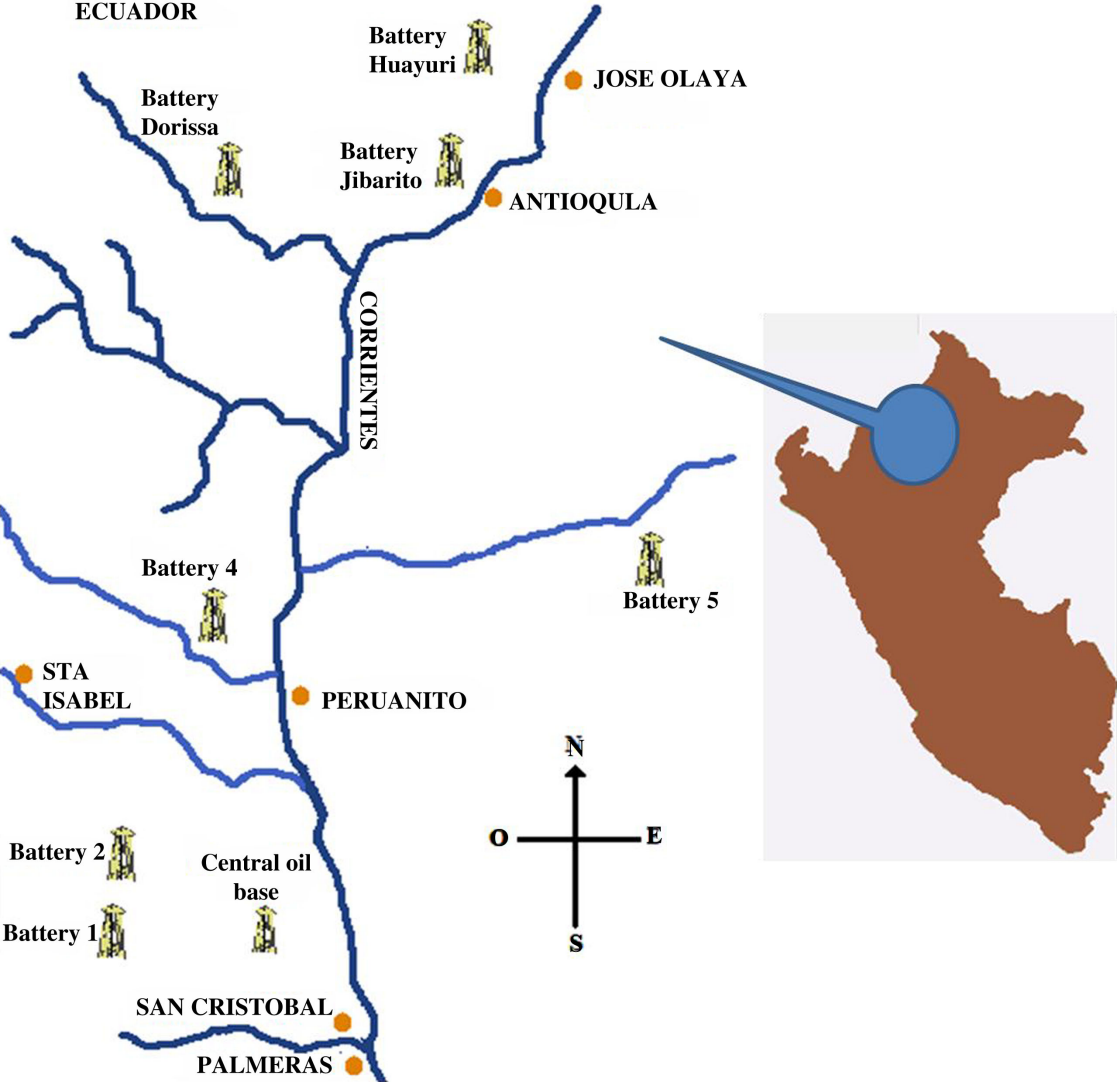
Map of the Corrientes River basin and indigenous communities.

The Corrientes river basin is located in the northeastern Peruvian Amazon, in the Loreto region. In 2008, the total population of the Corrientes river basin was estimated as 8,000, mainly formed by indigenous communities from the Achuar, Urarina, and Quichua ethnic groups and a minority of mestizos, with 52.0% under age 15 (16–18). This study was set in six communities (Fig. 1), selected based on their geographic location in relation to their history of oil exposure (number of oil spills nearby). In the upper river we selected Jose Olaya (population 0–17 years: 74) and Antioquia (population 0–17 years: 65), both located in close proximity (<2.5 km) to oil battery facilities. In the middle section, we selected Peruanito (population 0–17 years: 98), located 5 km downriver from the closest oil battery facility, and Santa Isabel de Copal (population 0–17 years: 146), located in the basin of the Copalyacu river (a tributary of the Corrientes), 42 km fluvial distance from the junction of the Corrientes and Copalyacu rivers, with no history of oil activity nearby. Downriver, we selected San Cristobal (population 0–17 years: 17) and Palmeras (population 0–17 years: 33), both proximate (<5 km) to oil battery facilities.

The study participants were all children and adolescents aged 0–17 years, whose families had lived in the area for the past 5 years and whose parents authorized their participation. For recruitment, all residents in the desired age range with these characteristics were sought.

### Data and samples collection

The fieldwork was carried out during November–December 2010 and included measurement of hemoglobin (Hb) levels, height and weight, and determination of BLLs conducted by nursing technicians, at the primary school of each community (especially arranged for this purpose).

Hb level was measured using a portable ß-hemoglobinometer (HemoCue, Lake Forest, CA, USA) from additional blood drops from capillary samples collected to test lead concentrations in blood. HemoCue standard controls were used for quality control. BLLs were determined using the Leadcare II Blood Lead Test System (ESA Biosciences, USA); a detailed explanation of this procedure can be found in Anticona et al. (19).

Height was measured with a standardized wooden board. Children younger than 24 months had their recumbent length measured using a pediatric length board. Weight was measured with a mechanical pediatric scale (maximum capacity 16 kg) for children younger than 2 years old and an upright scale (maximum capacity 160 kg) for the older ones.

Parents completed an interview-administered questionnaire that included demographic information; dwelling characteristics; water supply; parents’ occupation; history of employment at the oil company; and cooking and traditional practices. Specific information on the child included duration of breastfeeding and consumption (frequency and amount) of wild animal meat, fish, and other traditional foodstuffs. Trained local interviewers, who visited all households in the selected communities, collected this information.

### Data analysis

Hb levels were used to determine anemia status based on the cut-off points recommended by the World Health Organization (WHO) and used by Peru's national monitoring of nutritional indicators: <11.0 and 11.5 g/dL for the groups aged 6–59 months and 60–143 months, respectively, and 12 and 13 g/dL for the groups aged 144–179 months and older than 180 months, respectively (15, 20). Height and weight data were used to calculate z score values for height for age (H/A), weight for height (W/H), and weight for age (W/A), using Nutstat from Epi-Info software version 3.3.2 (Centers for Disease Control and Prevention (CDC), Atlanta, GA, USA). Those participants with a z score <−2 for H/A, W/A, and W/H were classified as presenting stunting, underweight, and wasting, respectively (21).

To compare our results with national statistics, we took stunting data as an indicator for chronic malnutrition. Stunting is considered the best epidemiological indicator for assessing chronic malnutrition because it reflects the accumulated, permanent, and long-term effects of deficiency in young child nutrition, including poor breastfeeding, complementary feeding practices, and repeated infections (22).

BLLs were categorized using the reference value of ≥5 µg/dL, which has been recently updated by the CDC due to the increasing evidence of various neurological impairments associated with low BLLs in children (23).

The variable ‘communities’ exposure to oil activity’ was classified into low, medium, and high, based on proximity of the community to an oil battery facility: no exposure (>20 km), Sta. Isabel; medium exposure (3–6 km), Peruanito, San Cristobal, and Palmeras; and high exposure (<2.5 km), Antioquia and Jose Olaya (13).

The data were analyzed using Stata version 10.0. Bivariate analysis was conducted using a Chi-square test to examine associations between nutritional indicators and single potential risk factors. Multivariate logistic regression analysis was conducted to examine the simultaneous impact of several factors on the probability to have anemia, stunting, or underweight. Wasting was excluded from the analysis due to its low frequency.

Some variables (water supply, parents’ occupation, history of employment at the oil company, cooking and traditional practices, duration of breastfeeding, and food consumption) were omitted from the analysis due to scarce variability.

In all of the analyses, participants were grouped into three age categories: early childhood (0–4 years), childhood (5–11 years), and adolescents (12–17 years), according to UNICEF classification (14).

### Ethics

The Research Ethics Committee of the Universidad Peruana Cayetano Heredia, Lima, approved this study. The consent process was conducted in Spanish and the native language of each community. The heads of family from the surveyed communities signed a consent form. The study findings were communicated to the communities in coordination with the indigenous organization FECONACO and the Regional Directorate of Health (DIRESA Loreto). Participants with elevated BLLs, malnutrition, or anemia were referred for medical evaluation to the Trompeteros Health Centre (Peru's Ministry of Health).

## Results

Data on Hb levels were collected from 330 participants aged 0–17 years representing a response rate of 76.2% (total population: 433). The non-participants were not present in their communities or dwellings during data collection. Some were attending secondary schools in other communities and some were accompanying their parents to perform fishing, agriculture, or hunting activities.

Overall, the prevalence of anemia was 51.0%. One case (a 5-year-old boy) was identified with severe anemia (Hb <7 g/dL). The bivariate analysis showed a significant difference in the proportion of anemia among age groups, with the highest prevalence in the 0–4 years group (*p*<0.05). No significant difference was found when comparing groups according to gender, community exposure to oil activity, consumption of river water, or BLLs ≥5 µg/dL (Table 1).

**Table 1:** Distribution of children and adolescents with anemia, stunting, wasting, and underweight, by potentially associated factors, Corrientes river basin, Peruvian Amazon, 2010

Gender	Anemia, *n* (%)	Stunting, *n* (%)	Underweight, *n* (%)	Wasting, *n* (%)
Boys	85 (54.8)	58 (52.7)	23 (20.9)	3 (3.0)
Girls	83 (47.4)	64 (47.4)	27 (20.0)	5 (5.0)
Age (years)				
0–4	74 (56.9)	40 (42.1)	30 (31.6)	6 (6.5)
5–11	68 (45.6)	58 (52.3)	18 (16.2)	2 (2.0)
12+	26 (50.9)	24 (61.5)	2 (5.1)	0
	*p<0.05*		*p<0.05*	
Community's exposure to oil activity				
No	74 (56.9)	55 (59.7)	23 (25.0)	1 (1.3)
Medium	56 (45.9)	44 (46.3)	19 (20.0)	4 (5.0)
High	38 (48.7)	23 (39.7)	8 (13.8)	3 (6.4)
Consumption of river water				
No	143 (50.5)	108 (51.7)	42 (20.1)	5 (3.0)
Yes	25 (53.2)	14 (38.9)	8 (22.2)	3 (9.0)
Blood lead levels (BLLs)				
BLLs<5 µg/dL	36 (48.6)	18 (36.7)	10 (20.4)	2 (4.4)
BLLs≥5 µg/dL	132 (51.6)	100 (53.5)	39 (20.8)	6 (4.1)
		*p<0.05*		
Total	168 (50.9)	122 (49.8)	50 (20.4)	8 (4.0)

Anthropometric data were collected from 245 participants (response rate of 56.6%). Missing information occurred because some participants did not go through all of the procedures. Overall, the prevalence of stunting and underweight were 50.0% and 20.0% respectively. There were eight cases (4.0%) of wasting (proxy of acute malnutrition). Bivariate analysis showed that stunting prevalence was similar when comparing age or gender groups (Table 1). However, the prevalence of underweight showed significant differences among age groups with the highest proportion in the youngest group (0–4 years) (*p*<0.05) (see Table 1).

In addition, no statistically significant association was found in the occurrence of stunting or underweight according to the child's consumption of river water or the level of exposure to the oil activity in the child's community of residence. However, the prevalence of stunting was significantly higher in the participants whose BLLs were ≥5 µg/dL (*p*<0.05).

In the logistic regression analysis, none of the assessed risk factors were associated with anemia or underweight. The groups aged 5–11 and ≥12 years had 1.9 times and 3.1 times higher risk of stunting than the group under five, respectively. In addition, participants with BLLs ≥5 µg/dL had twice the risk of stunting compared to those with lower BLLs (Table 2).

**Table 2:** Multivariate logistic models of OR and 95% CI for stunting in the study participants

Variable	Overall (n=236) OR (95%CI)
Age group	
0–4 years	1
5–11 years	1.9 (1.01–3.8)
12 +	3.1 (1.3–7.8)
Gender	
Girls	1.0
Boys	1.7 (0.9–2.9)
Reside in a community with high exposure to oil activity	
No	1
Yes	0.6 (0.4–1.0)
Underweight	
No	1
Yes	8.0 (3.7–20.4)
Anemia	
No	1
Yes	0.9 (0.5–1.5)
BLLs≥5 µg/dL	
No	1
Yes	2.0 (1.1–4.1)

## Discussion

Around half of the total studied population (51.0%) had anemia, with the highest frequency (56.9%) in children under five. These figures are higher than those estimated at the national level (39.0% for the group 0–17 years and 50.0% for the group 0–4 years) and for rural areas (42.6 for the group 0–17 years and 54.0% for the group 0–17 years) (14). However, higher rates have been reported in other populations in the Peruvian Amazon: 65.0% in children under five in the Amazonas region and 80.5% in children 7–14 months in the Loreto region (24, 25).

Compared to data from other countries of the Amazon River Basin, anemia is more frequent in Peru when considering school-age children and adolescents. For instance, 13.0% and 16.6% have been reported in children aged 0–10 years from the western Brazilian Amazon (26) and another group aged 5–14 years from the Ecuadorian Amazon (27), respectively. However, the prevalence is very similar when only looking at children under five. For instance, frequencies of 40.0% and 51.8% have been reported in children under five from the western Brazilian Amazon (26, 28).

When exploring risk factors for anemia, we found that being in the youngest group increased the risk significantly, which is similar to the findings in other studies of Amazon populations (25, 27–29).

Approximately half of the study population was also identified with stunting. Focusing on the group under five (data only available at the national level), stunting prevalence in the study population (42.1%) was higher than the national (18.0%) and regional level (22.0%) (7). Similar studies in other Peruvian Amazon indigenous communities from the Awajun ethnic group have reported lower rates ranging from 33.4% to 49.0% in children under five (24, 30, 31).

When comparing with data (collected in the last decade) from other countries in the Amazon River Basin, Peru again appears to be in a worse situation: 22.8% has been reported in Naporuna children under five from the Ecuadorian Amazon (32), 7.1% in children under five from the Brazilian Amazon (33), and 30.0% in children and adolescents aged 2–16 years from the Colombian Amazon (34).

Stunting was more prevalent in the oldest group. According to the literature, most stunting occurs in the first 2–3 years of life, and although there is biological potential for catch-up growth through adolescence, it is likely that individuals remaining in the same poor environments that caused them to become stunted in the first place will continue to be stunted into adulthood (35). Thus, our results might reflect a high frequency of stunting among older children and adolescents during their infancy, which has remained until the time of the study. We could not find any data from previous anthropometric measurements in the study population that could allow us to assess trends or changes across the different age groups.

A potential risk factor that we explored was elevated BLLs since this has been a particular health concern in the studied population. We found that the prevalence of stunting was higher in participants with BLLs ≥5 µg/dL.

The association between stunting and chronic lead exposure has been reported in previous studies (36). Similarly, elevated BLLs have been consistently associated with reduced growth velocity and lower stature in children with different blood lead concentrations, age ranges, and ethnic and socioeconomic characteristics (37–41).

The mechanism for how lead might directly depress children's growth is not clear. One hypothesis states that lead may interfere with vitamin D metabolism in children and thus disturb calcium metabolism, which may directly affect bone growth (37, 42, 43). Another hypothesis says that lead may displace calcium and interfere with its endocrine functions, including growth (42–44).

In addition, elevated BLLs have been associated with other socioeconomic factors that also increase the risk of child growth impairments (45), thus acting as confounders (37). Nevertheless, previous studies have shown significant association after adjusting for these covariates (37, 46). In fact, a threshold of 3 µg/dL has been recently proposed for lead effects on human growth (47).

Taking into account the large environmental pollution from oil extraction activities in the past 40 years (48), living in a clean environment (community of residence's exposure to oil activity) and access to safe water were also assessed as potential risk factors for stunting (49, 50). However, no association was found.

Particularly for Amazonian indigenous children, some authors have suggested that stunting could be attributed to a different growth potential and not necessarily to undernutrition, proposing that other cut-off points from those recommended by WHO should be used in nutritional surveys on indigenous communities (51). However, the majority argues that the high levels of stunting should be interpreted as reflective of their social conditions, which leads to poor growth on account of poor diets, frequent infections, and deficient child care, and not as a genetic trait (52).

Regarding underweight, the highest prevalence (30.0%) seen in children under five was much higher than the average for rural areas in Peru (9.0%) in the period 2003–2009 (53). In addition, we observed a positive association between underweight and stunting, suggesting that in this population, stunting could be attributed to a low intake of main nutrients.

This study had several limitations. First, we did not examine other risk factors for nutritional indicators that have been reported in other studies in the Amazon such as low maternal height, low birth weight, diarrhea episodes, and geohelminth infection (33).

Second, the small sample size and missing data from some participants might have contributed to finding a low variability of other factors explored and explain why we did not find other associations. Third, the cross-sectional design did not allow for the identification of the direction of the association between stunting and BLLs.

Finally, there were some limitations on samples and data collection. First, we used finger-prick blood samples to measure Hb and BLLs. This involves a procedure that can dilute the sample and thus introduce a systematic error into the results (54). Second, we used the LeadCare instrument for the analysis of BLLs. This method has been criticized because of its greater percentage of error and sensitivity to environmental conditions when compared to the gold standard method (graphite furnace atomic absorption). Nevertheless, the use of capillary blood samples and LeadCare has been supported for clinical evaluation and monitoring purposes in children (55, 56). Third, we used a questionnaire to collect information on risk factors. This may have introduced recall bias from respondents when answering about past events of the child's history and reporting bias, bearing in mind that respondents might have answered in ways that may be more socially acceptable. However, the following strategies were used to mitigate these biases: 1) interviewers visited every dwelling at a time when the majority of family members were present so that all could contribute to give comprehensive and reliable answers and 2) a local interpreter was available in case the majority of the household's members did not speak Spanish to ensure the questions were completely understood.

## Conclusions

Although improving nutritional status has been a global health priority in recent years, studies keep showing an extremely unfavorable scenario for indigenous children from the Amazon basin. In the communities of the Corrientes River Basin, half of the indigenous population aged 0–17 years was found to be affected with anemia and stunting. Anemia was more prevalent in the youngest age group (0–5 years), while stunting was found in the oldest age group (12–17 years). The association between stunting and BLLs might be attributed to a direct effect of lead on human growth. Also, poor nutrition and other socioeconomic-related factors may contribute to the simultaneous existence of stunting and elevated BLLs. Finally, these findings should be viewed as a marker of the need for tailored development programs and effective public health interventions for the indigenous populations of the Peruvian Amazon.

## References

[1] Pan American Health Organization (PAHO) (2010a). Health leaders endorse major effort to fight chronic malnutrition in Latin America and the Caribbean. http://new.paho.org/hq/index.php?option=com_content&view=article&id=7653%3Ahealth-leaders-endorse-major-effort-to-fight-chronic-malnutrivaluacition-in-latin-america-and-the-caribbean&catid=4243%3Ahsd0107x-cd-media-center&lang=es.

[2] PAHO (2010b). Anaemia in Latin America and the Caribbean, 2009: situation analysis, trends and implications for public health programming.

[3] Montenegro R, Stephens C (2006). Indigenous health in Latin America and the Caribbean. Lancet.

[4] Rivera J, Monterrubio E, González-Cossío T, García-Feregrino R, García-Guerra A, Sepúlveda-Amor J (2003). Nutritional status of indigenous children younger than five years of age in Mexico: results of a national probabilistic survey. Salud Publica Mex.

[5] Ruel MT, Menon P (2002). Child feeding practices are associated with child nutritional status in Latin America: innovative uses of the demographic and health surveys. J Nutr.

[6] Psacharopoulos G, Patrinos H (1994). Indigenous people and poverty in Latin America: an empirical analysis.

[7] Instituto Nacional de Estadística e Informática (INEI) (2009). Censos Nacionales 2007: IX de Población y VI de Vivienda. Resultados definitivos de las Comunidades Indígenas. Resumen Ejecutivo. Dirección Nacional de Censos y Encuestas.

[8] Instituto Nacional de Estadística e Informática (INEI) (2012). Encuesta Demográfica y de Salud Familiar 2012. http://proyectos.inei.gob.pe/web/biblioineipub/bancopub/Est/Lib1075/index.html.

[9] Benavides M, Mena M, Ponce C (2010). Estado de la Niñez Indígena en el Perú.

[10] Orta M, Napolitano D, MacLennan G, Callaghan C, Ciborowski S, Fabregas X (2007). Impacts of petroleum activities for the Achuar people of the Peruvian Amazon: summary of existing evidence and research gaps. Environ Res Lett.

[11] Dirección General de Salud Ambiental (DIGESA) (2010). Informe Evaluación de resultados del monitoreo del río Corrientes y toma de muestras biológicas, en la intervención realizada del 29 de junio al 15 de julio del 2005. http://www.minsa.gob.pe/portalMinsa/destacados/archivos/242/RIO%20CORRIENTES.pdf.

[12] Centro de Salud Ocupacional y Protección del Ambiente para la Salud (CENSOPAS) (2007). Informe técnico monitoreo biológico comunidades San Cristóbal y José Olaya, cuenca río Corrientes de Loreto, 19 al 26-07-2006.

[13] Anticona C (2012). Lead exposure in indigenous children of the Peruvian Amazon: seeking the hidden source, venturing into participatory research.

[14] United Nations Children's Fund (UNICEF) (2008). Situation of children in Peru. http://www.unicef.org/peru/spanish/Folleto_ing_correc_1.pdf.

[15] Ministerio de Salud del Peru (MINSA) (2009). Informe Resultados del Monitoreo Nacional de Indicadores Nutricionales (MONIN). Dirección ejecutiva de vigilancia alimentaria y nutricional.

[16] Proyecto Especial Plan Integral de Salud del Corrientes (PEPISCO) (2010). Censo 2010 Proyecto Especial Plan Integral de Salud del Corrientes.

[17] Ministerio de Salud del Peru (MINSA) (2006). Análisis de la situación de salud del pueblo Achuar. Serie análisis de situación de salud y tendencias.

[18] Pluspetrol (2006). Evaluación Técnica y Social de los Sistemas de Agua de las comunidades nativas de la Cuenca del Rio Corrientes.

[19] Anticona C, Bergdahl I, San Sebastian M (2012). Lead exposure among children from indigenous communities of the Peruvian Amazon basin. Rev Panam Salud Publica.

[20] World Health Organization (WHO) (2011). Haemoglobin concentrations for the diagnosis of anaemia and assessment of severity. Vitamin and Mineral Nutrition Information System. http://www.who.int/vmnis/indicators/haemoglobin.pdf.

[21] Dzieniszewski J, Jarosz M, Szczygie B, Marlicz K, Linke K, Lachowicz A (2005). Nutritional status of patients hospitalized in Poland. Eur J Clin Nutr.

[22] Panamerican Health Organization (PAHO) (2008). Malnutrition in infants and young children in Latin America and the Caribbean: achieving the Millennium Development Goals.

[23] Centers for Disease Control and Prevention (CDC) (2012). CDC response to advisory committee on childhood lead poisoning prevention recommendations in “low level lead exposure harms children: a renewed call of primary prevention” [document on the Internet]. http://www.cdc.gov/nceh/lead/ACCLPP/CDC_Response_Lead_Exposure_Recs.pdf.

[24] Gyorkos T, Maheu-Giroux M, Casapía M, Joseph S, Creed-Kanashiro H (2011). Stunting and helminth infection in early preschool-age children in a resource-poor community in the Amazon lowlands of Peru. Trans R Soc Trop Med Hyg.

[25] Huamán-Espino L, Valladares C (2006). Estado Nutricional y Características del Consumo Alimentario de la Población Aguaruna. Rev Peru Med Exp Salud Publica.

[26] Cardoso MA, Scopel KK, Muniz PT, Villamor E, Ferreira MU (2012). Underlying factors associated with anaemia in Amazonian children: a population-based, cross-sectional study. PLoS ONE.

[27] Quizhpe E, San Sebastian M, Hurtig AK, Llamas A (2003). Prevalence of anaemia in schoolchildren in the Amazon area of Ecuador. Rev Panam Salud Publica.

[28] De Souza OF, De Macedo LF, De Menezes CS, Santos de Araújo T, Torres P (2012). Prevalence and associated factors to anaemia in children. J Human Growth Develop.

[29] Oliveira C, Cardoso M, Araujo T, Muniz P (2011). Anaemia em crianças de 6 a 59 meses e fatores associados no Município de Jordão, Estado do Acre, Brasil. Cad Saúde Pública.

[30] Roche ML, Creed-Kanashiro HM, Tuesta I, Kuhnlein HV (2011). Infant and young child feeding in the Peruvian Amazon: the need to promote exclusive breastfeeding and nutrient-dense traditional complementary foods. Matern Child Nutr.

[31] Roche ML, Creed-Kanashiro HM, Tuesta I, Kuhnlein, HV (2007). Traditional food system provides dietary quality for the Awajún in the Peruvian Amazon. Ecol Food Nutr.

[32] Buitron D, Hurtig AK, San Sebastián M (2004). Estado nutricional en niños naporunas menores de cinco años en la Amazonía ecuatoriana. Rev Panam Salud Publica.

[33] Cobayashi F, Aparecida R, Hatzlhoffer B, Torres P, Augusto M (2013). Factors associated with stunting and overweight in Amazonian children: a population-based, cross-sectional study. Public Health Nutr.

[34] Elias L, Angulo E (2002). Desnutrición y su relación con parasitismo intestinal en niños de una población de la Amazonia colombiana. Biomédica.

[35] Martorell R, Khan LK, Schroeder DG (1994). Reversibility of stunting: epidemiological findings in children from developing countries. Eur J Clin Nutr.

[36] Bearer CF (2000). The special and unique vulnerability of children to environmental hazards. Neurotoxicology.

[37] Ballew C, Kettel L, Kaufmann R, Mokdad A, Miller D, Gunter EW (1999). Blood lead concentration and children's anthropometric dimensions in the Third National Health and Nutrition examination Survey (NHANES III), 1988–1994. J Pediatr.

[38] Schwartz J, Angle C, Pitcher H (1986). Relationship between childhood blood lead levels and stature. Pediatrics.

[39] Bithony WG (1989). Elevated lead levels in children with nonorganic failure to thrive. Pediatrics.

[40] Frisancho AR, Ryan AS (1991). Decreased stature associated with moderate blood lead concentrations in Mexican-American children. Am J Clin Nutr.

[41] Kafourou A, Touloumi G, Makropoulos V, Loutradi A, Papanagiotou A, Hatzakis A (1997). Effects of lead on the somatic growth of children. Arch Environ Health.

[42] Mahaffey KR (1983). Biotoxicity of lead: influence of various factors. Fed Proc.

[43] Rosen JF, Chesney FW, Hamstra AJ, DeLuca HF, Mahaffey KR (1983). Circulating calcitriol concentrations in health and disease. J Pediatr.

[44] Pounds JG, Long GJ, Rosen JF (1991). Cellular and molecular toxicity of lead in bone. Environ Health Prospect.

[45] Eveleth PB, Tanner JM (1990). Worldwide variation in human growth.

[46] Little BB, Spalding S, Walsh B, Keyes DC, Wainer J, Pickens S (1980). Blood lead levels and growth status among African American and Hispanic children in Dallas, Texas and 2002: Dallas Lead Project II. Ann Hum Biol.

[47] Schell LM, Denham MA, Stark AD, Parsons PJ, Schulte EE (2009). Growth of infants’ length, weight, head and arm circumferences in relation to low levels of blood lead measured serially. Am J Hum Biol.

[48] Earth Rights International (ERI) (2007). Racimos de Ungurahui, and Amazon Watch. A legacy of harm: occidental petroleum in indigenous territory in the Peruvian Amazon. http://www.earthrights.org/sites/default/files/publications/A-Legacy-of-Harm.pdf.

[49] Iunes RF, Monteiro CA, organizador (2000). Mudanças no cenário econômico. Velhos e novos males da saúde no Brasil.

[50] Santos RV (1993). Physical growth and nutritional status of Brazilian Indian populations. Cad Saude Publica.

[51] Holmes R, Sponsel LE (1995). Small is adaptive. Nutritional anthropology of native Amazonians. Indigenous peoples and the future of Amazonia.

[52] Bustos P, Amigo H, Muñoz SR, Martorell R (2001). Growth in indigenous and nonindigenous Chilean schoolchildren from 3 poverty strata. Am J Public Health.

[53] United Nations Children's Fund (UNICEF) (2010). Progress for children, achieving the MDGs with equity 9. http://www.unicef.org/protection/Progress_for_Children-No.9_EN_081710.pdf.

[54] Sari M, dePee S, Martini E, Herman S, Sugiatmi, Bloem MW (2001). Estimating the prevalence of anaemia: a comparison of three methods. Bull World Health Organ.

[55] Sobin C, Parisi N, Schaub T, De la Riva E (2011). A Bland-Altman comparison of the Lead Care^®^ System and inductively coupled plasma mass spectrometry for detecting low-level lead in child whole blood samples. J Med Toxicol.

[56] Centers for Disease Control and Prevention (CDC) (2004). Preventing lead exposure in young children: a housing-based approach to primary prevention of lead poisoning recommendations from the advisory committee on childhood lead. http://www.cdc.gov/nceh/lead/publications/PrimaryPreventionDocument.pdf.

